# Clinical Trials and Medical Care: Defining the Therapeutic Misconception

**DOI:** 10.1371/journal.pmed.0040324

**Published:** 2007-11-27

**Authors:** Gail E Henderson, Larry R Churchill, Arlene M Davis, Michele M Easter, Christine Grady, Steven Joffe, Nancy Kass, Nancy M. P King, Charles W Lidz, Franklin G Miller, Daniel K Nelson, Jeffrey Peppercorn, Barbra Bluestone Rothschild, Pamela Sankar, Benjamin S Wilfond, Catherine R Zimmer

## Abstract

A key component of informed consent to participate in medical research includes understanding that research is not the same as treatment.

## What Is Therapeutic Misconception?

For over three decades, bioethics scholarship and research ethics guidelines have identified concerns about the boundaries between research and standard clinical care [1,2]. Ethicists have argued that informed consent to participate in research should include clarification of the differences between these two activities [3–10]. In 1982, Appelbaum and colleagues reported on findings from interviews with patients with psychiatric disorders that documented failure to appreciate the difference between research and treatment, labeling the phenomenon “therapeutic misconception” (TM) [3].

Despite considerable empirical research on TM in the intervening years, a consistent definition has not emerged in the literature. Without such a definition, meaningful empirical work to measure and assess the prevalence of TM, or to test interventions to reduce it, is difficult to conduct. Progress is further impeded when studies use measures that reflect inconsistent definitions of research and clinical care, which are fundamental to the definition of TM.

Scholars who have contributed to this literature, including this paper's authors, met at the University of North Carolina at Chapel Hill in September 2005 to address the debate on defining TM. The workshop included a University of North Carolina team funded to study TM in early-phase gene transfer research (R01 HG 02087) [11–17] and others from the fields of medicine, oncology, public health, sociology, philosophy, anthropology, law, and bioethics. Following guidelines on scale development [18], we debated definitions based on the literature, evaluated questions that could be used in a TM scale, and participated in ongoing discussion during the following year. In this article, we summarize the controversies, propose a definition with specific dimensions, and describe how these dimensions can be operationalized to produce a valid measure of TM.

**Summary Points**

- A key component of informed consent to participate in medical research is the understanding that research is not the same as treatment.
- However, studies have found that some research participants do not appreciate important differences between research and treatment, a phenomenon called “therapeutic misconception.”
- A consistent definition of therapeutic misconception is missing from the literature, and this hinders attempts to define its prevalence or ways to reduce it.
- This paper proposes a new definition and describes how it can be operationalized.

## Defining Therapeutic Misconception

In Appelbaum and colleagues' study [3], the patients interviewed were enrolled in clinical trials that involved randomization, non-treatment control groups and placebos, and double-blind procedures. The researchers found that many trial participants were unaware of study design implications, especially random assignment to a control or comparison group, often believing that they were assigned a medication based on what was best for them, personally. The authors concluded that those patients who are trial participants and who do not adequately appreciate the purpose and methods of research studies are ill-equipped to evaluate risks and benefits of study participation, and may fail “to recognize how personal care may be compromised by research procedures” [19].

Confusion about the purpose of research is integral to most definitions of TM. According to Appelbaum and colleagues, TM occurs “when a research subject fails to appreciate the distinction between the imperatives of clinical research and of ordinary treatment, and therefore inaccurately attributes therapeutic intent to research procedures” [6]. In 2001, the National Bioethics Advisory Commission (NBAC) defined TM similarly, as “the belief that the purpose of a clinical trial is to benefit the individual patient rather than to gather data for the purpose of contributing to scientific knowledge” [5].

In its report, NBAC called attention to the important distinction between the purpose of research as a knowledge-generating activity and its broader consequences, which may include potential benefit from the intervention (direct benefit) or from other aspects of study participation (inclusion or collateral benefit) [20,21]. The report stated, “It is not a misconception to believe that participants probably will receive good clinical care during research. But it is a misconception to believe that the purpose of clinical trials is to administer treatment rather than to conduct research” [5]. Joffe and Miller [22], citing Levine [23] among others, point out that regardless of the potential for benefit to participants, research is always conducted in order to achieve scientific goals and contribute to generalizable knowledge [24]. Research participants who misunderstand this key point may not be able to make meaningful decisions to enroll in a clinical trial. Even participants who benefit, and who suffer little or no physical harm, may be wronged if they lack information essential to their decisions. Such non-physical harms or wrongs are called dignitary injuries (see a recent exchange [25,26] on the importance of this issue).

Some consider overestimation of clinical benefit from an experimental intervention, as well as underestimation of potential risk of harm [27], to be part of TM. Concern about the tendency to overestimate benefit has been prominent in discussions of early-phase cancer trials as well as other studies in which the likelihood of direct benefit is low, or in which the design (e.g., placebo-controlled trials) precludes direct benefit from an intervention for at least some participants [11,19,28–31]. Yet not all agree with the premise that overestimation of direct benefit from an experimental intervention is part of TM. Horng and Grady [32] argue that this phenomenon is different from and not integral to misunderstanding the nature and scientific intent of research. In addition, the extreme heterogeneity of clinical trial design makes generalizations about realistic expectation of direct benefit very difficult.

## Problems in Measuring TM

Since the original publication on TM [3], a number of empirical studies have explored the motivations, understandings, and expectations of patients who participate in research. Researchers have asked such questions as: Why do patients join a study? What is their understanding and recollection of the purpose of the research and particular aspects of study design? Can they differentiate the goals of research from those of clinical care? What are their expectations about the likelihood of direct benefit? These studies have used closed-ended questionnaires analyzed quantitatively [33], open-ended interviews analyzed qualitatively, or both [11,19]. Some studies included questions relevant to TM as part of a general investigation of ethical dilemmas in clinical trials, whether they set out to study TM [34] or not [31,35,36].

But with only a few exceptions [29,34,37], these studies did not use standardized questions and did not attempt to validate measures of TM or related constructs. Nor, with one notable exception [38], did they explore measurement issues such as how people understand the terms “research,” “treatment,” “experimental intervention,” or “study purpose.”

Studies of issues relevant to TM, which often use small, non-random samples, have documented misunderstanding among research participants related to characteristics such as older age, lower education, and the way in which information about the study is conveyed [11,19,33,34]. Only a few studies have included participants from different types of trials. For example, Henderson and colleagues [11] used a non-validated TM measure based on responses to interview questions about study purpose, reasons for participation, and expectations of direct benefit. They found that among participants in early-phase gene transfer research trials, those in trials for HIV and genetic disease had significantly lower scores on a composite TM scale than those in oncology or vascular disease trials. Nonetheless, because empirical investigations of TM have been undertaken without a consensus definition or consistent measures, the interpretation and comparability of such findings are in question, as are their implications for improving the informed consent process in clinical research.

One reason for uncertainty about the definition of TM is disagreement about what research participants ought to understand about the purpose of clinical research. There is consensus that they should understand that research has scientific goals. However, there are differences regarding what should be understood about therapeutic goals in clinical research.

Clinical investigators themselves hold different views on this issue [12,39]. In a recent survey of oncologists, Joffe and Weeks [7] found that 20% viewed the “main societal purpose of research” as ensuring state-of-the-art therapy for participants. This response may reflect underlying tension about the moral justification of research: that subjecting patients to potentially risky research is unethical unless clinical benefit is a legitimate research purpose [10]. In contrast, many bioethicists and clinical investigators find this view problematic because it confounds the purpose of research with its possible consequences [7,12,39,40].

## An Example of the Difficulty in Defining TM

Discordance in the literature on TM was reflected in our workshop discussions of the purpose of a research study. We considered whether the following question could be part of an instrument designed to measure TM:

“The purpose of the study is:

(1) Only to help patients enrolled in the study, or
(2) Both to help patients enrolled in the study and patients in the future, or
(3) Only to help patients in the future”

There was consensus that answer (1) is incorrect and reflects misunderstanding of the purpose of research studies, but there was disagreement about whether the correct answer was (2) or (3). Those who argued that (3) is the only correct response believe that the purpose of a trial is to further science and help future patients, not to help the patients enrolled in the study. According to this argument, the purpose of an experimental intervention is not to provide treatment (i.e., clinical trials are not treatment). The presence of concomitant clinical care and the potential for benefit associated with trial participation should not be confused with the fundamentally scientific goals of clinical trials.

In contrast, advocates of (2) as the correct response believe that helping patients enrolled in a study can be a legitimate *additional* study purpose. This may be because research and clinical care procedures and activities overlap, or because administration of an experimental agent is seen both as a means to learn about its safety and efficacy *and* as an appropriate therapeutic option. This conceptual debate reveals the difficulty of applying general assessments to trials that employ very different study designs. It is also relevant to recent empirical efforts to clarify whether or not participation in a clinical trial is associated with improved outcomes for participants [41–43].

## A New Definition of TM

Amid the controversies, there is consensus that the *defining* characteristic of research is to create generalizable knowledge through answering a scientific question. There is disagreement, however, regarding which elements of a trial could plausibly have a therapeutic purpose, whether additional therapeutic benefit ought to be counted as a *study purpose,* and whether overestimation of direct clinical benefit is part of TM.

To move beyond this impasse, we propose a consensus definition of TM that focuses on understanding the *defining* scientific purpose of research, irrespective of whether there are other reasonable goals. This definition acknowledges the important potential for clinical benefit and recognizes that opinions vary about whether and how clinical care and therapeutic purpose are combined with research. While the literature on TM has focused on patients who participate in research as the group most vulnerable to possible harm, Dresser [10] and others [7,12,44] have shown that TM is not limited to research participants. Thus our consensus definition is framed in terms general enough to be relevant to researchers, members of institutional review boards (research ethics committees), and others.

Finally, our definition (Box 1) does not include overestimation of the possible beneficial consequences of an experimental intervention. It is true that in many cases, TM may lead to overestimation of benefit, underestimation of risk of harm, or underappreciation of alternatives to participation. However, we argue that none of these results is a necessary consequence of TM; each could arise independently and coexist with an adequate understanding of the purpose of research.

**Box 1. Definition of Therapeutic Misconception**

Therapeutic misconception exists when individuals do not understand that the defining purpose of clinical research is to produce generalizable knowledge, regardless of whether the subjects enrolled in the trial may potentially benefit from the intervention under study or from other aspects of the clinical trial.

In order to develop specific questions to assess TM, we have identified five draft dimensions of research that individuals should understand, listed in Box 2. Using these dimensions, specific questionnaire items can be developed, tested for understandability, and ultimately combined to produce a composite measure of TM [18]. For example, questions based on the Scientific Purpose dimension, with agree–disagree or true–false responses, might include: “This study has been designed only to improve the health of the patients enrolled in it” and “If the experimental treatment is not effective, then the study is a failure.” Questions based on the Study Procedures dimension might include: “Procedures that have no benefit to patients in the study may still be done for scientific purposes” and “Every procedure in the study is designed only to help the patients in it.”

**Box 2. Five Draft Dimensions of Research that Should Be Understood by Trial Participants**

**Dimension 1. Scientific Purpose**

Clinical research is designed to produce generalizable knowledge and to answer questions about the safety and efficacy of intervention(s) under study in order to determine whether or not they may be useful for the care of future patients.

**Dimension 2. Study Procedures**

Participation in a trial may involve procedures or tests, in addition to the intervention(s) under study, that are intended only or primarily to generate scientific knowledge and that are otherwise not necessary for patient care.

**Dimension 3. Uncertainty**

For intervention(s) under study in clinical research, there often is less knowledge and more uncertainty about the risks and benefits to a population of trial participants than there is when a doctor offers a patient standard interventions.

**Dimension 4. Adherence to Protocol**

Administration of the intervention(s) under study is typically based on a strict protocol with defined dose, scheduling, and use or avoidance of concurrent medications, compared to administration of standard interventions.

**Dimension 5. Clinician as Investigator**

Clinicians who are in health care settings provide treatment; in a clinical trial setting, they are also investigating safety and efficacy of an intervention.

While our definition and draft dimensions are applicable across populations and studies, questions about TM should be tailored to the experiences of particular groups (e.g., participants or researchers). Within-group differences, such as those between patients participating in trials with different designs, may also require the use of tailored questions (e.g., trials only evaluating safety versus those with efficacy objectives). Discussion continues as we seek dimensions that apply to all clinical trials. Such discussion will require input from a variety of experts, including trial investigators, to be sure that these dimensions apply to diverse situations.

## Implications for Future Research

Refining the standard measurement of TM will provide a means to assess research understanding in different types of clinical trials and study populations. Such a tool can serve both to improve the protection of trial participants and refine the informed consent process, aiding accrual to clinical trials through a process that is both effective and efficient [15]. Historically, research on TM has been motivated by concern that participants may misunderstand aspects of trial care that lead them to make decisions incompatible with their true preferences and values. Though participants may recognize they are in a trial, failure to understand how care received during a trial can differ from standard care, and confusion over the purpose of these distinct activities, can compromise informed consent to research participation. While debate over other important aspects of informed consent is likely to continue, progress can be made in measuring TM by limiting our attention to those aspects that *clearly* interfere with trial participants' decision-making through failure to understand the defining nature and purpose of clinical research.

## Abbreviations

NBAC: National Bioethics Advisory Commission
TM: therapeutic misconception
